# Clinical manifestations in HTLV-1 infected individuals without HAM/TSP: Results of an 8 years cohort

**DOI:** 10.1186/1742-4690-11-S1-P3

**Published:** 2014-01-07

**Authors:** Davi Tanajura, Néviton Castro, Paulo Oliveira, Glória Orge, Anselmo Souza, Natália Barbosa, Silvane Santos, Edgar Carvalho

**Affiliations:** 1 Immunology Department, Universidade Federal da Bahia (UFBA), Salvador, Bahia, Brazil

## Introduction

HTLV-1 is an endemic virus in the northeast of Brazil and is related to HAM/TSP in up to 5% of infected individuals. Despite this low prevalence it is known that a higher number of carriers presents with clinical and neurological symptoms that can be sensory, motor, urinary, inflammatory or autonomic.

## Method

This is a cohort study with 440 HTLV-1 infected individuals in Salvador, Bahia. Symptoms were computed every year and incidence rates were calculated to each variable in data.

## Results

Applying international criteria 163 individuals had definite, possible or probable HAM/TSP and 266 were asymptomatic. The last was selected for analysis. Follow-up of at least one year was archived in 64 to 75% of patients, varying according to the studied variable. The symptoms incidence rate was 20.6% for hand numbness, 18.6% for feet numbness, 12.9% for nocturia, 12.6% for urgency and 13.3% for dry mouth. In the physical exam the incidence was 22.7% for gingivitis, 7.6% for legs hyperreflexia, 5.2% for legs paraparesis and 3.6% for Babinski sign. In annual applied EDSS scale the incidence rate of worsening 1 point was 13.4%. Cox regression was done with EDSS curves and identified proviral load and female gender as significant risk factors (ORs 3.36 [1-11.66] and 1.80 [1.09-2.97] respectively). Six patients developed HAM/TSP during the cohort.

## Conclusion

Developing of neurological symptoms or signs occurred in up to 30% of asymptomatic patients during 8 years follow-up. The estimated incidence was 12% for overactive bladder syndrome and 2.25% for definite HAM/TSP.

